# Influence of chronic pain on regional brain volume reduction in a general older Japanese population: a longitudinal imaging analysis from the Hisayama Study

**DOI:** 10.1093/braincomms/fcaf149

**Published:** 2025-04-16

**Authors:** Yuri Nakamura, Mao Shibata, Naoki Hirabayashi, Taro Nakazawa, Yoshihiko Furuta, Jun Hata, Masako Hosoi, Nobuyuki Sudo, Ken Yamaura, Toshiharu Ninomiya

**Affiliations:** Department of Epidemiology and Public Health, Graduate School of Medical Sciences, Kyushu University, Fukuoka 812-8582, Japan; Department of Anesthesiology and Critical Care Medicine, Graduate School of Medical Sciences, Kyushu University, Fukuoka 812-8582, Japan; Department of Epidemiology and Public Health, Graduate School of Medical Sciences, Kyushu University, Fukuoka 812-8582, Japan; Department of Psychosomatic Medicine, Graduate School of Medical Sciences, Kyushu University, Fukuoka 812-8582, Japan; Center for Cohort Studies, Graduate School of Medical Sciences, Kyushu University, Fukuoka 812-8582, Japan; Department of Epidemiology and Public Health, Graduate School of Medical Sciences, Kyushu University, Fukuoka 812-8582, Japan; Department of Psychosomatic Medicine, Graduate School of Medical Sciences, Kyushu University, Fukuoka 812-8582, Japan; Department of Epidemiology and Public Health, Graduate School of Medical Sciences, Kyushu University, Fukuoka 812-8582, Japan; Department of Neuropsychiatry, Graduate School of Medical Sciences, Kyushu University, Fukuoka 812-8582, Japan; Department of Epidemiology and Public Health, Graduate School of Medical Sciences, Kyushu University, Fukuoka 812-8582, Japan; Center for Cohort Studies, Graduate School of Medical Sciences, Kyushu University, Fukuoka 812-8582, Japan; Department of Medicine and Clinical Science, Graduate School of Medical Sciences, Kyushu University, Fukuoka 812-8582, Japan; Center for Cohort Studies, Graduate School of Medical Sciences, Kyushu University, Fukuoka 812-8582, Japan; Department of Health Care Administration and Management, Graduate School of Medical Sciences, Kyushu University, Fukuoka 812-8582, Japan; Department of Psychosomatic Medicine, Graduate School of Medical Sciences, Kyushu University, Fukuoka 812-8582, Japan; Department of Psychosomatic Medicine, Graduate School of Medical Sciences, Kyushu University, Fukuoka 812-8582, Japan; Department of Anesthesiology and Critical Care Medicine, Graduate School of Medical Sciences, Kyushu University, Fukuoka 812-8582, Japan; Department of Epidemiology and Public Health, Graduate School of Medical Sciences, Kyushu University, Fukuoka 812-8582, Japan; Center for Cohort Studies, Graduate School of Medical Sciences, Kyushu University, Fukuoka 812-8582, Japan

**Keywords:** chronic pain, pain-related brain regions, prospective cohort study, population-based

## Abstract

Longitudinal analyses of the influence of chronic pain on pain-related regional brain volumes in general populations are warranted. This prospective cohort study investigated the association between the presence of chronic pain at baseline and the subsequent changes in pain-related regional brain volumes among Japanese community-dwelling older residents. Participants aged 65 years or older who underwent brain magnetic resonance imaging (MRI) scans in both 2012 and 2017 were included. According to the presence or absence of chronic pain (defined as pain lasting for longer than 3 months) in 2012, participants were categorized into a ‘chronic pain’ group and ‘no chronic pain’ group. Region-of-interest analyses for the ventrolateral prefrontal cortex, dorsolateral prefrontal cortex, orbitofrontal cortex, postcentral gyrus, insular cortex, thalamus, anterior cingulate cortex, posterior cingulate cortex, amygdala and hippocampus were performed using FreeSurfer software. Whole-brain analysis was conducted by voxel-based morphometry. Rates of change in regional brain volume at 5 years after baseline were estimated using analysis of covariance. Among the 766 participants included in the FreeSurfer analysis, 444 (58%) were female and 287 (37%) were categorized into the chronic pain group. The results of FreeSurfer analysis showed that the chronic pain group had significantly greater decreases in regional volume in the postcentral gyrus (−2.187% in the chronic pain group versus −1.681% in the no chronic pain group, *P* = 0.01), thalamus (−4.400% versus −3.897%, *P* = 0.006), anterior cingulate cortex (−2.507% versus −1.941%, *P* = 0.004) and amygdala (−4.739% versus −4.022%, *P* = 0.03) compared to the no chronic pain group after adjusting for age, sex, education attainment, marital status, hypertension, diabetes, serum total cholesterol level, body mass index, current smoking, current drinking, regular exercise, cerebrovascular lesions on MRI, activities in daily living disability and depressive symptoms. Among the 730 participants included in the voxel-based morphometry analysis, 433 (59%) were female and 272 (37%) were categorized into the chronic pain group. The voxel-based morphometry analysis showed that the chronic pain group had a significantly greater regional volume decrease in the right anterior insula than the no chronic pain group. Our findings suggest that the presence of chronic pain at baseline is associated with a significantly greater decrease in the volume of pain-related brain regions at 5 years after baseline in community-dwelling older Japanese.

## Teaser

Nakamura *et al*. report that the presence of chronic pain at baseline is associated with a significantly greater decrease in the volume of the postcentral gyrus, thalamus, anterior cingulate cortex, amygdala, right hippocampus and right insula 5 years after baseline in 766 community-dwelling older Japanese.

## Graphical Abstract

**Graphical Abstract:**
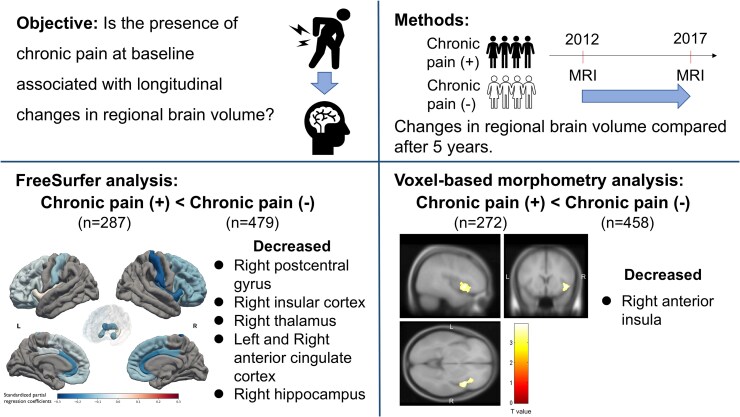

## Introduction

Chronic pain (CP) is a major cause of disabilities and is associated with burden at both the individual and global levels.^1,2^ In 2016, nociplastic pain was proposed as a new mechanistic category for CP.^3^ Nociplastic pain involves the concept of central sensitization—that is, plastic structural or functional alterations in the central nervous system.^4^ Pain information originating from the periphery is processed in the brain regions involved in the sensory-discriminative, affective-motivational and cognitive-evaluative components of pain. Studies indicate that CP causes changes in the brain that lead to abnormal pain processing and modulation.^1,5^ As for CP-related structural plasticity in the brain, several population-based and hospital-based studies have reported associations between CP and regional brain volume changes based on brain magnetic resonance imaging (MRI) scans. However, most of the previous population-based studies have had a cross-sectional design,^6-9^ and therefore the longitudinal changes caused by CP in the general population have not been sufficiently investigated. Moreover, because the previous studies examining the association between the presence of CP and longitudinal brain volume changes have been hospital-based, their participants have tended to have relatively severe pain and disability, limiting the applicability of their results to the general population.^10^ There is thus a need for longitudinal investigations into the influence of CP on regional brain volumes in general populations.

We herein conducted a prospective cohort study investigating the longitudinal association between the presence of CP at baseline and changes in the volume of pain-related brain regions 5 years later, using brain MRI data from Japanese community-dwelling older residents. For brain MRI data processing and analysis, we used two approaches widely adopted for this purpose: FreeSurfer software and voxel-based morphometry (VBM). FreeSurfer is a standard and well-validated tool for automated segmentation and volume estimation in region-of-interest (ROI) analysis,^11,12^ while VBM is a useful method for whole-brain analyses that include both cortical and subcortical brain structures and thus do not require setting ROIs.^13^ We conducted ROI analyses based on preceding studies using FreeSurfer.^7,8^ In addition, to investigate whether the associations were present in regions beyond the selected ROIs, we performed whole-brain analyses using VBM. We also examined the dose–response relationship between pain severity and brain volume changes. Finally, we addressed a limitation of previous population-based studies—namely, although hemispheric specialization in pain processing has been reported, differences between left and right regional brain volumes have not been adequately investigated. Accordingly, we examined the lateral heterogeneity in the association of CP and brain volume changes.

## Materials and methods

### Study population

The Hisayama Study is an ongoing population-based observational study conducted in the town of Hisayama, a suburb of the Fukuoka metropolitan area in southern Japan.^14^ In addition to annual health examinations since 1961, screening surveys for cognitive function and activities of daily living have been conducted on the town residents aged ≥65 years every 5–7 years since 1985.^15^ In 2012 and 2017, brain MRI scans were performed as a part of the survey. Supplementary Fig. 1 provides a detailed flow chart of the participant selection. In 2012, a total of 1906 residents aged ≥65 years (93.6% of the town's population in this age group) participated in the screening survey. Among 1342 participants who underwent brain MRI examination, we excluded 1 participant who did not consent to the use of their 2012 MRI data, 115 participants who were deceased by 31 March 2017, 284 participants who did not undergo brain MRI examination in 2017, 3 participants who did not consent to the use of their 2017 MRI data, 17 participants without a three-dimensional T1-weighted (3DT1) scan in 2012 or 2017, 44 participants with a history of dementia and 96 participants without available data for CP. The remaining 782 participants were included in the longitudinal brain MRI analysis. Among these participants, in order to maximize the sample size for each approach, we excluded participants with inappropriate data for the two methods of brain MRI analysis as follows: (i) for the FreeSurfer analyses, we excluded 16 participants who had MRI data unsuitable for analysis, resulting in a group of 766 participants (322 male and 444 female); and (ii) for the VBM analyses, we excluded 3 participants with metal artefacts, 1 with excessive motion artefact and 48 with cortical or cerebellar infarcts on MRI, as these might have affected the assessment of brain volume by VBM. The final number of participants in the VBM analyses was 730 (297 male and 433 female). Among the total of 777 participants, 719 participants (94% of the FreeSurfer analysis participants and 98% of the VBM analysis participants) were included in both analyses.

The present study was approved by the Kyushu University Institutional Review Board for Clinical Research (approval no. 23061-03). Written informed consent was obtained from all participants according to the Declaration of Helsinki.

### Definition of chronic pain

The exposure variable in this study was the presence of CP in 2012. CP was defined as pain lasting for longer than 3 months.^1,16^ Participants filled out a questionnaire at the 2012 survey that asked whether they had any pain lasting more than 3 months. Based on their response, participants were then categorized into a ‘CP’ group or ‘no CP (NCP)’ group. Participants with CP were also asked to indicate their average pain intensity over the previous week on a 100-mm visual analogue scale (VAS).

### Assessment of brain volume

In 2012 and 2017, brain MRI examinations were performed at the screening survey venue using a 1.5-Tesla scanner (Intera Pulsar; Philips Medical Systems, Best, the Netherlands) with a multichannel head coil, including 3DT1 images, conventional T1- and T2-weighted images, fluid-attenuated inversion recovery images, T2*-weighted images and magnetic resonance angiography. To ensure the consistency of brain MRI scans at the two time points, T1-weighted three-dimensional images were acquired in the sagittal plane using the same parameters for all participants both in 2012 and 2017: repetition time 8.5 ms, echo time 4.0 ms, inversion time 1000 ms, flip angle 8°, field of view 240 mm, acquisition matrix 192 × 192, slice thickness 1.2 mm and number of excitations 1.

For ROI analyses, 3DT1 image processing including segmentation and volume measurements of cortical and subcortical brain structures and intracranial volume (ICV) were performed automatically with the longitudinal processing stream in FreeSurfer [version 6.0.0; Harvard University, Boston, MA (https://surfer.nmr.mgh.harvard.edu)].^17^ Cortical parcellation was based on the Desikan–Killiany atlas.^13^ All segmentation results were visually double-checked for accuracy by seven study members, including an expert stroke neurologist and expert psychiatrists, according to the Enhancing Neuro Imaging Genetics through Meta Analysis (ENIGMA) Cortical Quality Control Protocol 2.0.^18^ The scans detected as having inaccurate segmentation were removed from the analysis. Regional brain volumes were calculated as a percentage of ICV (measured by FreeSurfer longitudinal processing stream) with adjustment for head size, and were taken as the sum of the left and right sides. Volume changes in each brain region at 5 years after baseline were calculated as follows: ([regional brain volume in 2017/ICV − regional brain volume in 2012/ICV]/regional brain volume in 2012/ICV) × 100 = ([regional brain volume in 2017 − regional brain volume in 2012]/regional brain volume in 2012) × 100 (%). We also calculated volume changes in each left and right brain region separately. To minimize the alpha error by multiple comparisons, we selected the following 10 brain regions as the ROIs for the present study referring to preceding studies^7,8^: the ventrolateral prefrontal cortex, dorsolateral prefrontal cortex, orbitofrontal cortex, postcentral gyrus, insular cortex, thalamus, anterior cingulate cortex, posterior cingulate cortex, amygdala and hippocampus. These regions were selected based on a systematic review.^7^

For whole-brain analysis using VBM, MRI data were processed using the ‘longitudinal model for large changes' of the Computational Anatomy Toolbox [CAT12, version 12.8; University of Jena, Jena, Germany (https://dbm.neuro.uni-jena.de/cat/)] in SPM12 [University College London, London, UK (https://www.fil.ion.ucl.ac.uk/spm/)] running under MATLAB (MathWorks, Natick, MA). All MRI scans of VBM analysis participants were assigned image quality ratings of over 60% by CAT12, and were thus considered suitable for analysis.^19^ The default parameters were used except that affine regularization was performed with the International Consortium for Brain Mapping template for East Asian brains. The 3DT1 images underwent intra-subject realignment, bias correction, segmentation and normalization (normalization was estimated for the mean image of both time points and then applied to both images). Segmented grey matter images were modulated to compensate for the volumetric effects of expansion or shrinking that occur in spatial normalization. The images were smoothed with an 8 mm full-width at half-maximum isotropic Gaussian kernel. ICV was calculated as the sum of the grey matter, white matter and cerebrospinal fluid volumes. Grey matter difference maps were calculated by subtracting each participant's smoothed grey matter map in 2012 from that in 2017 using the ‘ImCalc’ function in SPM12.

### Definition of covariates

Information on education attainment, marital status, medication for hypertension and diabetes, smoking, alcohol intake and exercise was collected by using a self-administered questionnaire in 2012. Depressive symptoms were assessed using the Geriatric Depression Scale short form (GDS) and defined as a GDS score ≥ 6.^20^ Activities in daily living (ADL) were assessed by the Barthel index, and ADL disability was defined as a Barthel index ≤ 95.^21,22^ Body mass index (BMI, kg/m^2^) was calculated from height and weight measured in light clothing without shoes. Hypertension was defined as systolic blood pressure ≥ 140 mmHg or diastolic blood pressure ≥ 90 mmHg based on the mean of three blood pressure measurements in a sitting position, or current use of antihypertensive agents.^23^ Plasma glucose levels were measured by the hexokinase method, and diabetes mellitus was defined as a fasting plasma glucose level of ≥7.0 mmol/L (126 mg/dL) or a 2-h post-loaded or casual glucose level of ≥11.1 mmol/L (200 mg/dL) in accordance with the 2006 World Health Organization criteria,^24^ or current use of glucose-lowering agents. Serum total cholesterol level was measured enzymatically. Cerebrovascular lesions were defined as brain infarction or haemorrhage observed on MRI in 2012 regardless of the presence or absence of neurological symptoms. The detailed definition was previously described.^25^

### Statistical analysis

To describe the baseline characteristics of the NCP and CP groups, age- and sex-adjusted mean values and frequencies of covariates were calculated by using analysis of covariance (ANCOVA) and logistic regression analysis, respectively. We also compared the age- and sex-adjusted mean volume of each brain ROI at baseline (expressed as a percentage of ICV) between the two groups using ANCOVA. For FreeSurfer analysis, the mean values of volume changes in each brain region with 95% confidence intervals in the NCP and CP groups were estimated using ANCOVA, adjusting for potential clinical confounding factors.^8^ Two models were evaluated as follows: model 1, adjusted for age and sex; and model 2, adjusted for age, sex, education attainment (≤9 years or >9 years), marital status (currently with or currently without a partner), hypertension, diabetes, serum total cholesterol level, BMI, current smoking, current drinking, regular exercise (engaging in sports or other physical exercise including recreational walking for ≥3 times a week during leisure time or not), cerebrovascular lesions on MRI, ADL disability and depressive symptoms. To elucidate the dose–response relationship between the CP intensity at baseline and the regional brain volume changes, we dichotomized the CP group at the median (39.4 mm) of VAS into a low pain-intensity group and high pain-intensity group, and compared the results with those for the NCP group. Trends across the groups of CP intensity were tested by using a relevant analysis of covariance model including the CP intensity categories as an ordinal variable (i.e. 0 for NCP, 1 for low pain intensity and 2 for high pain intensity). One participant was excluded from the analysis due to missing data on CP intensity. To assess the hemispheric specialization in the association between CP and regional brain volume change, we calculated the volume changes in each left and right brain region separately. The heterogeneity in the association between CP and the left and corresponding right regional brain volume change was tested using a generalized estimating equation. To deal with the alpha-error accompanying multiple comparisons, we performed false discovery rate (FDR) correction.^26^ An FDR *Q*-value of <0.10 was defined as sufficient to verify the multiple comparisons for the neuroimaging study.^27^ In addition, to allow direct comparisons between different brain regions, we performed *Z*-score standardization for the volume change rates of each brain region and calculated standardized partial regression coefficients of each regional volume change according to the presence of CP using linear regression models.^7^ The analyses described above were performed with the SAS software package version 9.4 (SAS Institute, Cary, NC). Two-sided values of *P* < 0.05 were considered statistically significant.

For VBM analysis, we hypothesized that regional grey matter volume would decrease more prominently in the CP group than the NCP group over the 5 years after baseline measurements. Statistical parametric mapping for the difference image was performed using ANCOVA between the NCP and CP groups after adjustment for the above-mentioned covariates in model 2 and ICV (measured by CAT 12). We applied explicit masking using a grey matter template included in the CAT12 Toolbox (Template_4_GS.nii) with grey matter values of >0.05. To correct for multiple comparisons, statistical parametric mappings were thresholded at uncorrected *P* < 0.001 at the voxel level combined with a familywise error-corrected *P* < 0.05 at the cluster level.

## Results

For FreeSurfer analysis, the median interval between the 2012 and 2017 brain MRI scans for all participants was 5.1 years [interquartile range (IQR): 5.0–5.1 years]. In the 2012 survey, 287 participants (37.5%) had CP. The median intensity of VAS was 39.4 mm (IQR: 23.1–57.4 mm). The age- and sex-adjusted participant characteristics according to the presence or absence of CP in 2012 are summarized in Table 1. Compared to those of the NCP group, the mean age, the mean BMI and the proportion of education attainment ≤ 9 years were significantly higher in the CP group. No significant difference in the age- and sex-adjusted mean volume of each brain ROI was observed at baseline (Supplementary Table 1). The characteristics of the NCP and CP group participants in the VBM analysis were similar to those of NCP and CP group participants in the FreeSurfer analysis, as described in Supplementary Table 2.

**Table 1:** Age- and sex-adjusted baseline characteristics of the FreeSurfer analysis participants according to the presence or absence of chronic pain (*n* = 766), 2012

Variable	Overall	Chronic pain status		*P*-value
		No chronic pain	Chronic pain	
	(*n* = 766)	(*n* = 479)	(*n* = 287)	
Age, mean (SE), years^a^	71.9 (0.2)	**71.6 (0.2)**	**72.4 (0.3)**	**0.04**
Female, %^b^	58.0	55.9	61.5	0.13
Education attainment, ≤9 years, %	31.1	**28.4**	**35.7**	**0.04**
Marital status, without partner, %	22.0	22.9	20.3	0.40
Hypertension, %	67.0	66.0	68.6	0.45
Diabetes mellitus, %	22.1	20.9	24.3	0.27
Serum total cholesterol, mean (SE), mg/dL	201.0 (1.2)	200.8 (1.5)	201.4 (1.9)	0.83
Body mass index, mean (SE), kg/m^2^	23.2 (0.1)	**23.0 (0.1)**	**23.6 (0.2)**	**0.009**
Current smoking, %	5.2	4.3	6.6	0.11
Current drinking, %	44.1	44.6	43.2	0.75
Regular exercise, %	43.2	44.7	40.7	0.29
Cerebrovascular lesions on MRI, %	28.5	27.1	30.7	0.30
ADL disability, %	1.4	1.0	2.1	0.14
Depressive symptoms, %	10.5	9.8	11.8	0.40

SE, standard error; MRI, magnetic resonance imaging; ADL, activities of daily living. Values where significant differences were observed are shown in bold (*P* < 0.05). ^a^Age was sex-adjusted. ^b^Proportion of female was age-adjusted.

Table 2 shows the age- and sex-adjusted (model 1) and multivariable-adjusted (model 2) mean values of volume change in each brain region at 5 years after baseline according to the presence or absence of CP in 2012. The 5-year volume reductions in the postcentral gyrus, thalamus, anterior cingulate cortex and amygdala in the CP group were significantly greater than those in the NCP group after adjusting for potential clinical confounding factors. The observed associations in these regions remained significant after FDR correction.

**Table 2:** Adjusted mean values of volume changes in each brain region at 5 years after baseline according to the presence or absence of chronic pain (*n* = 766), 2012–17

Brain region	Adjusted mean values (95% CIs) of volume changes, %		*P*-value	*q*-Value of FDR correction
	No chronic pain (*n* = 479)	Chronic pain (*n* = 287)		
Ventrolateral prefrontal cortex, %				
Model 1	−2.786 (−3.004 to −2.568)	−3.042 (−3.324 to −2.760)	0.16	0.22
Model 2	−2.797 (−3.017 to −2.578)	−3.023 (−3.307 to −2.738)	0.22	0.27
Dorsolateral prefrontal cortex, %				
Model 1	−2.630 (−2.887 to −2.373)	−2.907 (−3.239 to −2.575)	0.20	0.22
Model 2	−2.641 (−2.899 to −2.382)	−2.889 (−3.224 to −2.554)	0.25	0.28
Orbitofrontal cortex, %				
Model 1	−2.662 (−2.895 to −2.430)	−2.925 (−3.225 to −2.624)	0.18	0.22
Model 2	−2.669 (−2.904 to −2.435)	−2.913 (−3.217 to −2.609)	0.22	0.30
Postcentral gyrus, %				
Model 1	**−1.670 (−1.907 to −1.433)**	**−2.205 (−2.511 to −1.898)**	**0.007**	**0.03**
Model 2	**−1.681 (−1.919 to −1.442)**	**−2.187 (−2.496 to −1.878)**	**0.01**	**0.04**
Insular cortex, %				
Model 1	−1.997 (−2.232 to −1.763)	−2.349 (−2.653 to −2.046)	0.07	0.16
Model 2	−2.013 (−2.249 to −1.778)	−2.323 (−2.628 to −2.018)	0.12	0.22
Thalamus, %				
Model 1	**−3.884 (−4.100 to −3.668)**	**−4.421 (−4.701 to −4.142)**	**0.003**	**0.02**
Model 2	**−3.897 (−4.115 to −3.679)**	**−4.400 (−4.682 to −4.117)**	**0.006**	**0.03**
Anterior cingulate cortex, %				
Model 1	**−1.934 (−2.167 to −1.702)**	**−2.518 (−2.818 to −2.217)**	**0.003**	**0.03**
Model 2	**−1.941 (−2.175 to −1.706)**	**−2.507 (−2.811 to −2.203)**	**0.004**	**0.046**
Posterior cingulate cortex, %				
Model 1	−2.168 (−2.447 to −1.890)	−2.575 (−2.935 to −2.215)	0.08	0.13
Model 2	−2.186 (−2.465 to −1.906)	−2.546 (−2.908 to −2.183)	0.13	0.20
Amygdala, %				
Model 1	**−4.033 (−4.430 to −3.635)**	**−4.721 (−5.235 to −4.207)**	**0.04**	0.11
Model 2	**−4.022 (−4.421 to −3.623)**	**−4.739 (−5.256 to −4.221)**	**0.03**	**0.09**
Hippocampus, %				
Model 1	−5.696 (−6.018 to −5.374)	−6.008 (−6.425 to −5.591)	0.25	0.25
Model 2	−5.703 (−6.026 to −5.380)	−5.996 (−6.415 to −5.577)	0.28	0.28
Total brain volume, %				
Model 1	−3.354 (−3.488 to −3.220)	−3.554 (−3.727 to −3.381)	0.07	0.14
Model 2	−3.363 (−3.496 to −3.229)	−3.540 (−3.714 to −3.367)	0.11	0.25

CIs, confidence intervals; FDR, false discovery rate. Values where significant differences were observed are shown in bold (*P* < 0.05). Values were calculated as follows: ([regional brain volume in 2017 − regional brain volume in 2012]/regional brain volume in 2012) × 100 (%). Regional brain volumes were calculated as the sum of the left and right sides. Model 1: adjusted for age and sex. Model 2: adjusted for age, sex, education attainment, marital status, hypertension, diabetes, serum total cholesterol level, body mass index, current smoking, current drinking, regular exercise, cerebrovascular lesions on magnetic resonance imaging, activities of daily living disability and depressive symptoms. An FDR *Q*-value < 0.10 was defined as sufficient to verify the multiple comparisons.

Table 3 shows the dose–response relation between the CP intensity at baseline and the volume changes in each brain region. As the pain intensity increased, the magnitude of the volume reduction in the anterior cingulate cortex and amygdala also tended to increase after adjusting for potential clinical confounding factors (model 2, *P* for trend = 0.006 in the anterior cingulate cortex and 0.04 in the amygdala); however, in the amygdala, this trend was attenuated after FDR correction. The detailed results including age- and sex-adjusted mean values are shown in Supplementary Table 3.

**Table 3:** Multivariable-adjusted mean values of volume changes in each brain region at 5 years after baseline according to the pain intensity of chronic pain (*n* = 765), 2012–17

Brain region	Multivariable-adjusted mean values (95% CIs) of volume changes, %			*P* for trend	*q*-Value of FDR correction
	No chronic pain	Chronic pain, VAS ≤ 39.4 mm	Chronic pain, VAS > 39.4 mm		
	(*n* = 479)	(*n* = 143)	(*n* = 143)		
Ventrolateral prefrontal cortex, %	−2.798 (−3.017 to −2.578)	−3.169 (−3.572 to −2.767)	−2.881 (−3.285 to −2.476)	0.45	0.55
Dorsolateral prefrontal cortex, %	−2.641 (−2.900 to −2.383)	−3.070 (−3.544 to −2.596)	−2.713 (−3.190 to −2.237)	0.51	0.56
Orbitofrontal cortex, %	−2.670 (−2.904 to −2.435)	−3.108 (−3.538 to −2.677)	−2.717 (−3.149 to −2.285)	0.52	0.52
Postcentral gyrus, %	−1.681 (−1.919 to −1.443)	−2.350 (−2.787 to −1.912)	−2.027 (−2.466 to −1.588)	0.05	0.15
Insular cortex, %	−2.015 (−2.250 to −1.779)	−2.414 (−2.845 to −1.982)	−2.244 (−2.678 to −1.811)	0.21	0.46
Thalamus, %	**−3.897 (−4.115 to −3.679)**	**−4.411 (−4.811 to −4.011)**	**−4.376 (−4.778 to −3.974)**	**0.01**	**0.08**
Anterior cingulate cortex, %	**−1.941 (−2.175 to −1.706)**	**−2.450 (−2.880 to −2.020)**	**−2.565 (−2.998 to −2.133)**	**0.006**	**0.06**
Posterior cingulate cortex, %	−2.187 (−2.466 to −1.907)	−2.718 (−3.231 to −2.205)	−2.374 (−2.889 to −1.858)	0.30	0.54
Amygdala, %	**−4.024 (−4.423 to −3.624)**	**−4.681 (−5.414 to −3.949)**	**−4.793 (−5.529 to −4.057)**	**0.04**	0.15
Hippocampus, %	−5.705 (−6.028 to −5.381)	−6.008 (−6.601 to −5.415)	−5.974 (−6.569 to −5.378)	0.35	0.55
Total brain volume, %	−3.363 (−3.496 to −3.229)	−3.661 (−3.906 to −3.416)	−3.415 (−3.661 to −3.169)	0.37	0.51

CIs, confidence intervals; VAS, visual analogue scale; FDR, false discovery rate. Values where significant differences were observed are shown in bold (*P* < 0.05). Values were calculated as follows: ([regional brain volume in 2017 − regional brain volume in 2012]/regional brain volume in 2012) × 100 (%). Regional brain volumes were calculated as the sum of the left and right sides. Values were adjusted for age, sex, education attainment, marital status, hypertension, diabetes, serum total cholesterol level, body mass index, current smoking, current drinking, regular exercise, cerebrovascular lesions on magnetic resonance imaging, activities of daily living disability and depressive symptoms. An FDR *Q*-value of <0.10 was defined as sufficient to verify the multiple comparisons.

**Figure 1:**
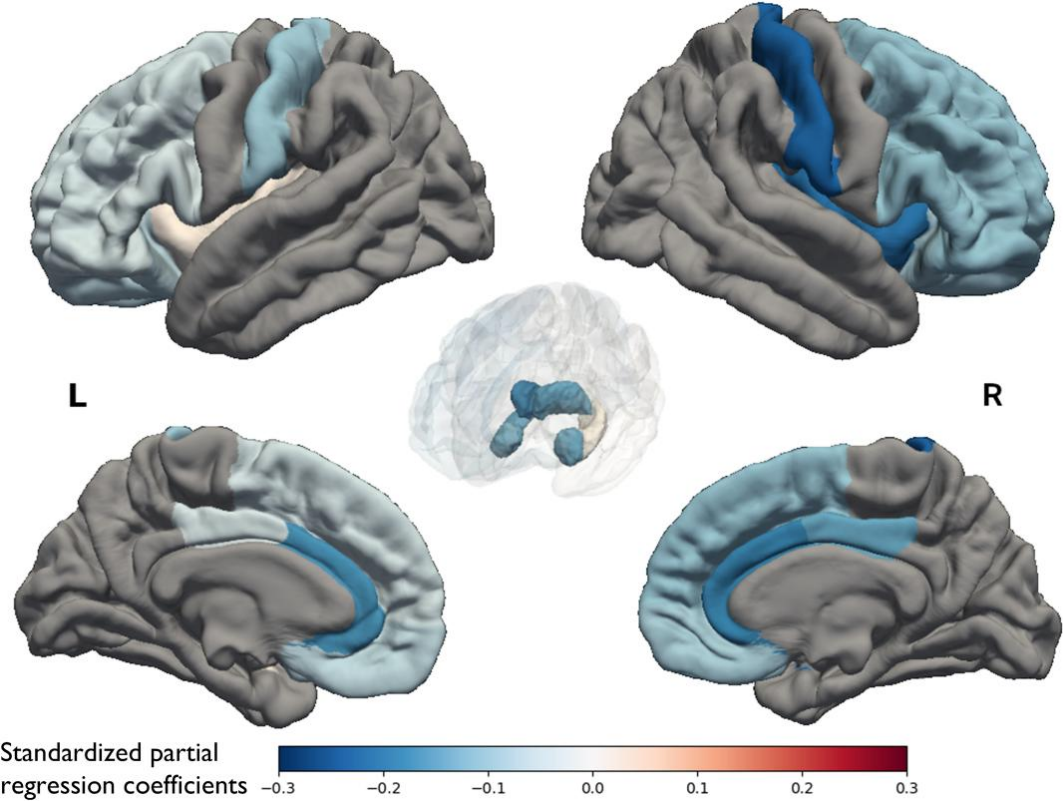
**Association between the presence of chronic pain at baseline and changes in grey matter volume 5 years later based on FreeSurfer analysis (number of participants = 766).** A linear regression model was used to visualize the standardized partial regression coefficients of each left and right region of interest for a chronic pain group (number of participants = 287) versus a no chronic pain group (number of participants = 479). In the chronic pain group, the volume reductions in the right postcentral gyrus, right insular cortex, right thalamus, left and right anterior cingulate cortex and right hippocampus were significantly greater than those in the no chronic pain group. Results were adjusted for age, sex, education attainment, marital status, hypertension, diabetes, serum total cholesterol level, body mass index, current smoking, current drinking, regular exercise, cerebrovascular lesions on MRI, activities in daily living disability, depressive symptoms and intracranial volume. L, left; R, right.

Next, we compared the volume changes in each left and right brain region separately between the CP and NCP groups (Table 4). In the CP group, the volume reductions in the right postcentral gyrus, right insular cortex, right thalamus, left and right anterior cingulate cortex and right hippocampus were significantly greater than those in the NCP group after adjusting for potential confounding factors (model 2). The magnitudes of the association between CP at baseline and the volume reductions in the insular cortex and hippocampus were stronger on the right side than on the left side (*P* for heterogeneity = 0.004 and =0.03, respectively). The association with CP observed in the right hippocampus and the heterogeneity found in the hippocampus did not remain significant after FDR correction. Figure 1 visualizes the standardized partial regression coefficients of each left and right ROI for the CP group compared to the NCP group as a reference. The standardized partial regression coefficients for the analyses shown in Tables 2–4 are given in Supplementary Tables 4–6.

**Table 4:** Multivariable-adjusted mean values of volume changes in each left and right brain region at 5 years after baseline according to the presence or absence of chronic pain (*n* = 766), 2012–17

Brain region	Multivariable-adjusted mean values (95% CIs) of volume changes, %					
	No chronic pain (*n* = 479)	Chronic pain (*n* = 287)	*P*-value	*q*-Value of FDR correction	*P* for hetero.	*q*-Value of FDR correction
Ventrolateral prefrontal cortex, %						
Left	−2.823 (−3.074 to −2.573)	−2.968 (−3.293 to −2.644)	0.49	0.58	0.44	0.73
Right	−2.762 (−2.993 to −2.530)	−3.053 (−3.353 to −2.753)	0.13	0.24		
Dorsolateral prefrontal cortex, %						
Left	−2.637 (−2.907 to −2.367)	−2.809 (−3.159 to −2.459)	0.45	0.56	0.14	0.35
Right	−2.640 (−2.903 to −2.376)	−2.965 (−3.307 to −2.624)	0.14	0.24		
Orbitofrontal cortex, %						
Left	−2.799 (−3.049 to −2.548)	−3.009 (−3.334 to −2.684)	0.32	0.42	0.67	0.83
Right	−2.530 (−2.791 to −2.269)	−2.808 (−3.147 to −2.469)	0.21	0.29		
Postcentral gyrus, %						
Left	−1.700 (−1.966 to −1.434)	−2.004 (−2.348 to −1.659)	0.17	0.27	0.07	0.22
Right	**−1.630 (−1.903 to −1.357)**	**−2.352 (−2.706 to −1.998)**	**0.002**	**0.03**		
Insular cortex, %						
Left	−2.019 (−2.303 to −1.735)	−1.969 (−2.338 to −1.601)	0.83	**0.88**	**0.004**	**0.04**
Right	**−1.989 (−2.257 to −1.721)**	**−2.653 (−3.001 to −2.306)**	**0.003**	**0.03**		
Thalamus, %						
Left	−3.974 (−4.239 to −3.708)	−4.407 (−4.751 to −4.062)	0.05	0.13	0.69	0.76
Right	**−3.804 (−4.063 to −3.545)**	**−4.379 (−4.714 to −4.043)**	**0.008**	**0.06**		
Anterior cingulate cortex, %						
Left	**−1.794 (−2.082 to −1.506)**	**−2.346 (−2.720 to −1.972)**	**0.02**	**0.09**	0.72	0.72
Right	**−2.001 (−2.284 to −1.719)**	**−2.591 (−2.958 to −2.225)**	**0.01**	**0.07**		
Posterior cingulate cortex, %						
Left	−2.134 (−2.446 to −1.821)	−2.308 (−2.714 to −1.903)	0.51	0.56	0.16	0.32
Right	−2.179 (−2.535 to −1.823)	−2.765 (−3.227 to −2.303)	0.05	0.14		
Amygdala, %						
Left	−4.224 (−4.726 to −3.723)	−5.033 (−5.683 to −4.383)	0.06	0.12	0.59	0.84
Right	−3.849 (−4.275 to −3.424)	−4.465 (−5.017 to −3.913)	0.09	0.17		
Hippocampus, %						
Left	−5.826 (−6.184 to −5.468)	−5.791 (−6.255 to −5.326)	0.91	0.91	**0.03**	0.14
Right	**−5.587 (−5.938 to −5.236)**	**−6.211 (−6.666 to −5.756)**	**0.03**	0.12		

CIs, confidence intervals; FDR, false discovery rate; hetero., heterogeneity. Values where significant differences were observed are shown in bold (*P* < 0.05). Values were calculated as follows: ([regional brain volume in 2017 − regional brain volume in 2012]/regional brain volume in 2012) × 100 (%). Values were adjusted for age, sex, education attainment, marital status, hypertension, diabetes, serum total cholesterol level, body mass index, current smoking, current drinking, regular exercise, cerebrovascular lesions on magnetic resonance imaging, activities of daily living disability and depressive symptoms. An FDR *Q*-value of <0.10 was defined as sufficient to verify the multiple comparisons.

**Figure 2:**
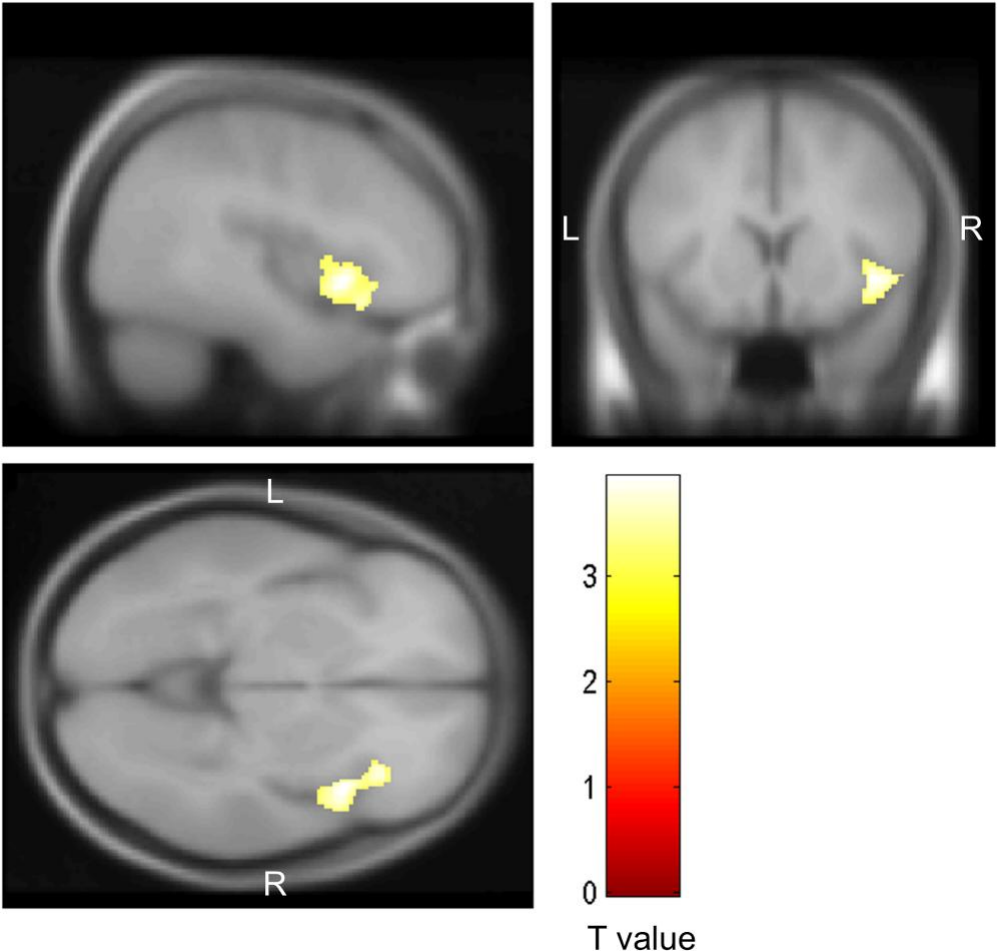
**Association between the presence of chronic pain at baseline and changes in grey matter volume 5 years later based on voxel-based morphometry analysis (number of participants = 730).** Brain regions where the presence of chronic pain was negatively correlated with volume changes, based on analysis of covariance, are coloured. The right anterior insula was the main region where the grey matter volume decrease was greater in the chronic pain group (number of participants = 272) than the no chronic pain group (number of participants = 458). Results were adjusted for age, sex, education attainment, marital status, hypertension, diabetes, serum total cholesterol level, body mass index, current smoking, current drinking, regular exercise, cerebrovascular lesions on MRI, activities in daily living disability, depressive symptoms and intracranial volume. Slice images are displayed in keeping with the neurologic conventions (the right hemisphere is displayed on the right of the figure). The colour intensity in the yellow–red heat map represents the *T*-statistic value of each voxel between the presence of chronic pain and changes in regional grey matter volume. Yellow indicates a higher *T*-statistic value (i.e. a stronger inverse correlation) compared with red. L, left; R, right.

Finally, in the whole-brain analyses using VBM, the CP group showed significantly greater volume reduction in the right anterior insula than the NCP group (Fig. 2).

## Discussion

The present prospective longitudinal study demonstrated that the presence of CP at baseline was associated with significantly greater volume reduction in the postcentral gyrus, anterior cingulate cortex, thalamus and amygdala at 5 years after baseline in Japanese community-dwelling older residents. As the pain intensity increased, the magnitude of volume reductions in the anterior cingulate cortex and amygdala tended to be greater. When the analysis was performed separately for the left and right sides of the brain, the presence of CP at baseline was associated with significantly greater volume reductions in the right postcentral gyrus, right insular cortex, right thalamus, left and right anterior cingulate cortex and right hippocampus. The whole-brain analysis using VBM showed that the presence of CP at baseline was associated with a significantly greater volume decrease in the right anterior insula.

The brain regions where significant associations were seen in the present study have key roles in pain processing. The thalamus functions as a gateway to relay pain-related information in the lateral and medial nociceptive systems.^28^ The lateral nociceptive system projects to the postcentral gyrus, also known as the primary somatosensory cortex, where stimulus localization is discriminated.^28^ The anterior cingulate cortex, projected by the medial nociceptive system, represents the affective component of pain.^28^ The pain-associated information ascends the spino-parabrachio-amygdaloid pathway, and the amygdala contributes to the formation of pain-related emotional memory and responses.^29^ It is reasonable that the chronic input of pain stimuli to these brain regions leads to greater volume changes. Several previous cross-sectional studies have evaluated the association between CP and regional brain volume in general populations.^6-9^ A study from Germany including 543 participants reported that chronic back pain was associated with decreased regional grey matter in the prefrontal cortex and the anterior insula; the finding of a decrease in the prefrontal cortex diverged from our present study, where we found no association between chronic pain and volume change in this region.^6^ Another study of 3376 participants in the Netherlands found that chronic joint pain in female was associated with lower hippocampus volume and lower total, temporal lobe, and frontal lobe grey matter volume, while no significant associations were observed in male, which is in accordance with our present results for the hippocampus but not with those for the frontal lobe.^7^ A study based on 11 106 UK Biobank participants showed that a chronic localized back pain group and chronic widespread pain group had lower postcentral gyrus grey matter volume compared with pain-free controls, which corresponded with our present finding of volume reduction in the postcentral gyrus in participants with CP.^9^ In a previous cross-sectional analysis of 1106 participants in the Hisayama Study, we found that the chronic low back pain group had significantly lower volumes of the ventrolateral prefrontal cortex, dorsolateral prefrontal cortex, posterior cingulate cortex and amygdala than the no CP group, which is consistent with our present findings for the amygdala but not the prefrontal cortex regions.^8^ On the other hand, previous studies comparing the magnitude of the longitudinal changes in regional brain volumes in individuals with and without CP have been primarily hospital-based,^10,30-32^ and population-based studies have been limited. The present study revealed that the volumes of the postcentral gyrus, amygdala, and hippocampus were significantly reduced longitudinally in the CP group compared to the NCP group, which would support the results from the previous population-based cross-sectional studies. In addition, we found significant brain volume reductions in the thalamus and anterior cingulate cortex, in agreement with the findings of hospital-based studies.^30-34^ However, unlike the previous cross-sectional studies, our present analysis revealed no significant difference in volume reduction in the prefrontal cortex regions between the CP and NCP groups. Since there is a possible bidirectional relationship between CP and brain volume,^35^ with pain potentially causing volume changes and volume changes enhancing pain, the difference in results between the present and previous studies may imply that the volume alterations of the prefrontal cortex regions contribute to the development and maintenance of CP.^6^

When we examined the left and right regional brain volumes separately by ROI analysis with FreeSurfer, we found that the magnitude of the association between CP at baseline and the volume reduction in the insular cortex was greater on the right side than the left side. In addition, the whole-brain analysis by VBM showed that CP was associated with significantly greater volume reduction in the right anterior insula. The results from the FreeSurfer and VBM analyses suggest that the right anterior insula is a significant brain area that is likely to exhibit a larger decrease in regional volume in association with CP at baseline. The anterior insula is associated with the cognitive-affective dimension of pain, while the posterior insula is associated with the sensory dimension of pain.^36,37^ In a cross-sectional, hospital-based study, patients with CP showed a significant volume decrease in the right anterior insular cortex compared with healthy controls, and also showed decreasing connectivity between the right anterior insular cortex and the reward system in association with increasing pain catastrophizing and depression scores.^38^ Taken together, these results suggest that the eventual decrease in right anterior insula volume in individuals with CP contributes to the affective and cognitive modulations of pain perception. Moreover, the greater volume reduction on the right side than on the left side in association with CP was also seen in the hippocampus. In line with our findings, two previous cross-sectional studies among community-dwelling older adults demonstrated that CP was associated with smaller volumes of the right and total hippocampus, but not the left hippocampus.^39,40^ Although hemispheric specialization in pain processing has been reported, information on the existence of pain-related asymmetry has been conflicting.^41^ Further studies are needed to describe the relationship between CP and asymmetric volume changes in pain-related brain regions.

In the present study, VBM revealed a significant association with CP only in the right anterior insula, whereas FreeSurfer showed significant associations with CP in multiple brain regions. Limitations of VBM, including potential misregistration and reduced accuracy of region location due to spatial normalization, may have contributed to our inability to replicate some of the associations found in FreeSurfer.^13,42^ Furthermore, FreeSurfer investigates entire brain volumes of each brain structure on a much larger scale than the voxel-by-voxel (1.5 mm^3^) investigation in VBM. This could have made FreeSurfer more sensitive to diffuse and subtle volume changes across a given brain structure.^42^ The dose–response relationship between VAS and volume reduction in the amygdala, the association between CP and volume reduction in the right hippocampus and the lateral heterogeneity seen in the hippocampus were not significant after FDR correction. This may have been due to insufficient statistical power, although we also cannot rule out the possibility of alpha-errors due to multiple comparisons.

The mechanisms underlying the association between CP and changes in pain-related regional brain volumes remain to be elucidated. Possible explanations include CP-associated neurodegeneration, decrease in cell size, neural or glial cell apoptosis, synaptic loss, decrease in spine density, decrease in blood flow, or changes in interstitial fluid.^32,33,43^ In addition, neuroinflammation involved by CP-activated brain glial cells is thought to be one of the plausible mechanisms.^4,44^ Microglia are a type of glial cells that function as the resident macrophages in the central nerve system, and play a crucial role in maintaining the brain homeostasis under normal conditions. However, excessive microglial activation leads to detrimental effects on brain cells and extracellular matrix structures via the release of inflammatory cytokines.^45,46^ Microglial cell activations are observed in pain-related brain regions such as the thalamus, anterior cingulate cortex and amygdala after peripheral nerve injury in mice.^47-49^ Glial cell activation in the thalamus and postcentral gyrus is also seen in patients with chronic low back pain.^50^ These were the same brain regions where we observed an association with the presence of CP in the present study. Synaptic remodelling and hyperexcitability, or reduction of the dendritic complexity, caused by activated microglial cells may contribute to the decreased volume in pain-related brain regions.^51,52^ Regarding other possible mechanisms, stress, fear, or depression, which are commonly complicated by CP, also could contribute to brain volume loss.^53^ As the associations seen in the present study stayed significant after adjusting for depression, the presence of CP itself appears to be associated with larger regional brain volume loss independently of depression. Additionally, since brain volume loss has been reported in opioid users,^54^ the influence of opioids as a pain medication on pain-induced brain volume reduction should be considered. However, we could not investigate the influence of opioids in the present study because only four of our participants used opioids. We therefore consider that the influence of opioid intake on the associations seen in our study participants, if any, would be very subtle. Interestingly, some studies have reported that changes in the volume or thickness of pain-related brain regions were reversed after arthroplasty in patients with hip or knee osteoarthritis,^30,32,55^and after effective treatment by spine surgery or facet joint block in patients with chronic low back pain.^31^ Furthermore, an increase in cerebral grey matter has been reported after cognitive behavioural therapy in patients with CP.^56^ These reports suggest that the mechanism by which the volumes of pain-related brain regions decline in the presence of CP is at least partially reversible for some pain sites or pathologies, or under certain treatment regimens. Pain relief through the treatment of CP is expected to reverse the pain-related regional brain volume and restore appropriate brain functions involved in recognition, emotion and decision-making.^31^

The present study examined the association between the presence of CP at baseline as an exposure variable and the longitudinal change of pain-related regional brain volumes based on brain MRI data at two time points in a relatively large number of older individuals in the general population. Moreover, we adjusted for a wide range of factors that could confound the association between the presence of CP and the regional brain volume changes. We also used two different approaches, i.e. FreeSurfer and VBM analysis. In addition to these strengths, several limitations should be noted. (1) The validity of the CP definition was limited. Since the definition of CP was based on self-reported information, it could not incorporate the results of detailed clinical evaluation. (2) Selection bias was present. Brain MRI examinations were performed only on participants who were able to come to the screening survey venue, which led to a selection bias for relatively health-conscious participants with preserved health and ADL. (3) The generalizability was limited. The results cannot be readily generalized to populations with severe impairments of health or ADL, or to other populations. (4) The spatial resolution may have been low. We used a 1.5-Tesla scanner to conduct brain MRI examinations at the screening survey venue. This could have resulted in lower spatial resolution compared to a 3-Tesla scanner. (5) Residual confounding cannot be ruled out. Unmeasured factors, such as information on stress, fear, or the details of CP therapies, may have led to residual confounding. (6) Potential associations between the course of CP (resolved, persisted or evolved) or functional impairment and the observed brain changes could not be elucidated.

In conclusion, the present study demonstrated that the presence of CP at baseline was associated with significantly greater reductions in pain-related regional brain volumes 5 years after baseline in Japanese community-dwelling older residents. These findings imply that pain relief through CP treatment may reverse the pain-related decline in regional brain volume, and also restore the brain functions involved in pain processing, in hospital-based patients with CP as well as in older individuals in the general population.

## Supplementary Material

fcaf149_Supplementary_Data

## Data Availability

The datasets used in the current study are not publicly available because they contain confidential clinical data on the study participants. However, the data are available on reasonable request and with the permission of the Principal Investigator of the Hisayama Study, To.N. (Department of Epidemiology and Public Health, Graduate School of Medical Sciences, Kyushu University, Fukuoka, Japan).
