# Supplementary material for: Influence of chronic pain on regional brain volume reduction in a general older Japanese population: a longitudinal imaging analysis from the Hisayama Study
Source: Brain Commun. 2025 Apr 16;7(2):fcaf149. doi: 10.1093/braincomms/fcaf149 (PMC12018798; doi:10.1093/braincomms/fcaf149)
Supplement: fcaf149_Supplementary_Data [file fcaf149_supplementary_data.pdf]

Supplementary Fig. 1. Flow chart of the study participant selection.

Abbreviations: MRI, magnetic resonance imaging; 3DT1, 3-dimensional T1-weighted; VBM, voxel-based morphometry.

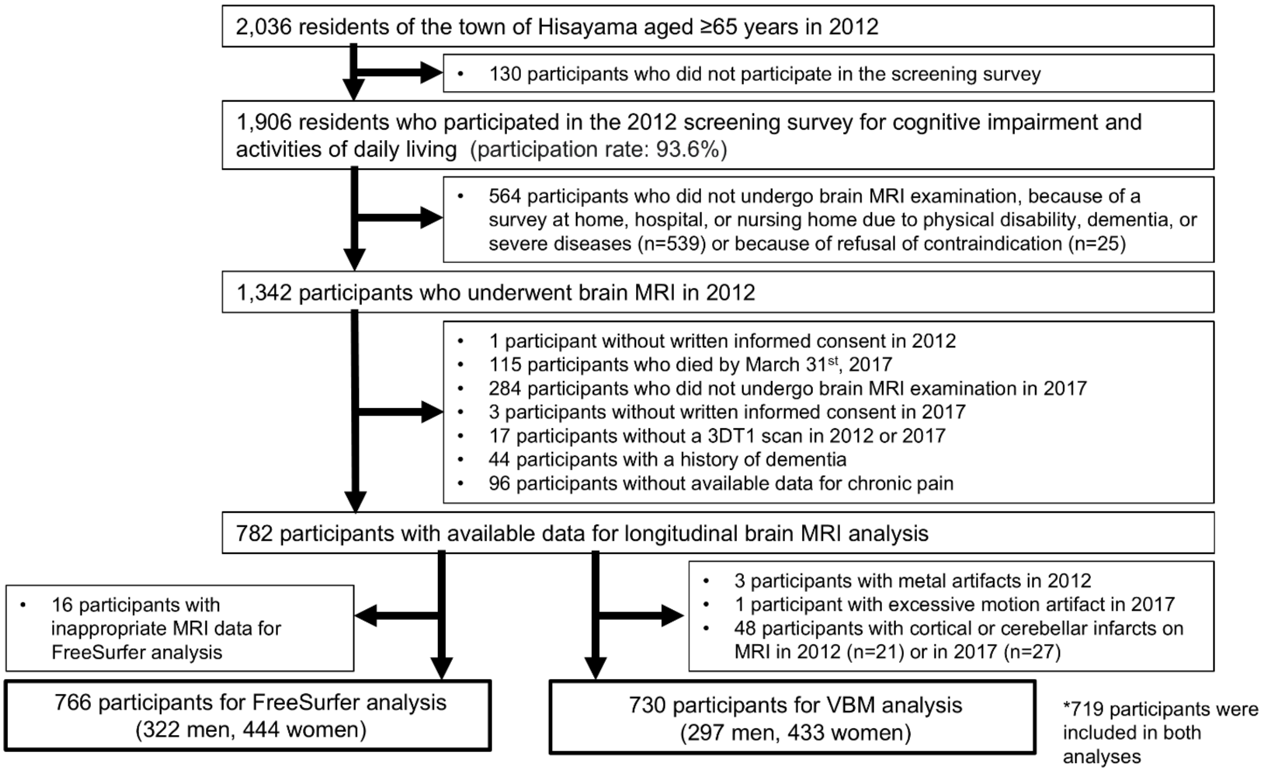

Supplementary Table 1. Age- and sex-adjusted regional brain volumes (expressed as a percentage of ICV) of the FreeSurfer analysis participants at baseline according to the presence or absence of chronic pain (*n* = 766), 2012

Brain region	Overall	Chronic pain status		*P* value
		No chronic pain	Chronic pain	
	(*n* = 766)	(*n* = 479)	(*n* = 287)	
Ventrolateral prefrontal cortex, mean (SE), %ICV	1.268 (0.004)	1.271 (0.006)	1.263 (0.007)	0.41
Dorsolateral prefrontal cortex, mean (SE), %ICV	5.112 (0.012)	5.122 (0.016)	5.096 (0.020)	0.30
Orbitofrontal cortex, mean (SE), %ICV	1.690 (0.004)	1.692 (0.005)	1.686 (0.007)	0.50
Postcentral gyrus, mean (SE), %ICV	1.144 (0.004)	1.143 (0.005)	1.147 (0.006)	0.59
Insular cortex, mean (SE), %ICV	0.914 (0.003)	0.915 (0.003)	0.912 (0.004)	0.58
Thalamus, mean (SE), %ICV	0.857 (0.002)	0.860 (0.003)	0.852 (0.004)	0.07
Anterior cingulate cortex, mean (SE), %ICV	0.480 (0.002)	0.479 (0.003)	0.483 (0.004)	0.38
Posterior cingulate cortex, mean (SE), %ICV	0.386 (0.001)	0.387 (0.002)	0.383 (0.002)	0.16
Amygdala, mean (SE), %ICV	0.184 (0.001)	0.185 (0.001)	0.183 (0.001)	0.19
Hippocampus, mean (SE), %ICV	0.492 (0.002)	0.494 (0.002)	0.488 (0.003)	0.08
Total brain volume, mean (SE), %ICV	68.490 (0.104)	68.584 (0.132)	68.334 (0.170)	0.25

Abbreviations: ICV, intracranial volume; SE, standard error.
Values were calculated as follows: ([left regional brain volume in 2012 + right regional brain volume in 2012] / ICV) × 100 (%).

Supplementary Table 2. Age- and sex-adjusted baseline characteristics of the VBM analysis participants according to the presence or absence of chronic pain (*n* = 730), 2012

Variable	Overall	Chronic pain status		*P* value
		No chronic pain	Chronic pain	
	(*n* = 730)	(*n* = 458)	(*n* = 272)	
Age, mean (SE), years[a]	71.8 (0.2)	71.5 (0.2)	72.3 (0.3)	0.05
Women, %[b]	59.3	57.4	62.6	0.16
Education attainment, ≤9 years, %	31.2	**28.2**	**36.4**	**0.02**
Marital status, without partner, %	22.4	23.1	21.2	0.56
Hypertension, %	66.1	65.7	66.8	0.76
Diabetes, %	21.8	21.1	23.0	0.54
Serum total cholesterol, mean (SE), mg/dL	201.6 (1.2)	201.2 (1.5)	202.1 (2.0)	0.73
Body mass index, mean (SE), kg/m^2	23.2 (0.1)	**23.0 (0.2)**	**23.6 (0.2)**	**0.008**
Current smoking, %	5.2	4.3	6.6	0.12
Current drinking, %	43.7	44.5	42.4	0.63
Regular exercise, %	44.1	46.1	40.7	0.16
Cerebrovascular lesions on MRI, %	26.4	25.8	27.3	0.67
ADL disability, %	1.4	0.9	2.3	0.08
Depressive symptoms, %	10.9	10.0	12.4	0.32

Abbreviations: SE, standard error; MRI, magnetic resonance imaging; ADL, activities of daily living.
Values where significant differences were observed are shown in bold ($p<0.05$).
a) Age was sex-adjusted.
b) Proportion of women was age-adjusted.

Supplementary Table 3. Adjusted mean values of volume changes in each brain region at 5 years after baseline according to the pain intensity of chronic pain (*n* = 765), 2012-2017

Brain region	Adjusted mean values (95% CIs) of volume changes, %			*P* for trend	*q* value of FDR correction
	No chronic pain (*n* = 479)	Chronic pain, VAS ≤39.4 mm (*n* = 143)	Chronic pain, VAS >39.4mm (*n* = 143)		
**Ventrolateral prefrontal cortex, %**					
Model 1	-2.786 (-3.004 to -2.568)	-3.195 (-3.595 to -2.795)	-2.895 (-3.294 to -2.496)	0.37	0.45
Model 2	-2.798 (-3.017 to -2.578)	-3.169 (-3.572 to -2.767)	-2.881 (-3.285 to -2.476)	0.45	0.55
**Dorsolateral prefrontal cortex, %**					
Model 1	-2.630 (-2.887 to -2.373)	-3.075 (-3.546 to -2.604)	-2.747 (-3.217 to -2.277)	0.41	0.45
Model 2	-2.641 (-2.900 to -2.383)	-3.070 (-3.544 to -2.596)	-2.713 (-3.190 to -2.237)	0.51	0.56
**Orbitofrontal cortex, %**					
Model 1	-2.662 (-2.895 to -2.429)	-3.120 (-3.546 to -2.694)	-2.730 (-3.155 to -2.305)	0.46	0.46
Model 2	-2.670 (-2.904 to -2.435)	-3.108 (-3.538 to -2.677)	-2.717 (-3.149 to -2.285)	0.52	0.52
**Postcentral gyrus, %**					
Model 1	**-1.670 (-1.908 to -1.433)**	**-2.367 (-2.802 to -1.932)**	**-2.045 (-2.479 to -1.611)**	**0.04**	0.14
Model 2	-1.681 (-1.919 to -1.443)	-2.350 (-2.787 to -1.912)	-2.027 (-2.466 to -1.588)	0.05	0.15
**Insular cortex, %**					
Model 1	-1.998 (-2.233 to -1.763)	-2.449 (-2.879 to -2.020)	-2.265 (-2.694 to -1.836)	0.15	0.32
Model 2	-2.015 (-2.250 to -1.779)	-2.414 (-2.845 to -1.982)	-2.244 (-2.678 to -1.811)	0.21	0.46
**Thalamus, %**					
Model 1	**-3.884 (-4.101 to -3.668)**	**-4.416 (-4.812 to -4.019)**	**-4.413 (-4.809 to -4.018)**	**0.007**	**0.04**
Model 2	**-3.897 (-4.115 to -3.679)**	**-4.411 (-4.811 to -4.011)**	**-4.376 (-4.778 to -3.974)**	**0.01**	**0.08**
**Anterior cingulate cortex, %**					
Model 1	**-1.935 (-2.167 to -1.702)**	**-2.473 (-2.899 to -2.047)**	**-2.563 (-2.988 to -2.137)**	**0.004**	**0.045**
Model 2	**-1.941 (-2.175 to -1.706)**	**-2.450 (-2.880 to -2.020)**	**-2.565 (-2.998 to -2.133)**	**0.006**	**0.06**
**Posterior cingulate cortex, %**					
Model 1	-2.168 (-2.447 to -1.890)	-2.767 (-3.278 to -2.257)	-2.386 (-2.895 to -1.877)	0.23	0.42
Model 2	-2.187 (-2.466 to -1.907)	-2.718 (-3.231 to -2.205)	-2.374 (-2.889 to -1.858)	0.30	0.54
**Amygdala, %**					
Model 1	-4.034 (-4.432 to -3.636)	-4.712 (-5.442 to -3.983)	-4.729 (-5.457 to -4.001)	0.06	0.15
Model 2	**-4.024 (-4.423 to -3.624)**	**-4.681 (-5.414 to -3.949)**	**-4.793 (-5.529 to -4.057)**	**0.04**	0.15
**Hippocampus, %**					

Model 1	-5.697 (-6.020 to -5.375)	-6.045 (-6.636 to -5.454)	-5.961 (-6.551 to -5.371)	0.33	0.46
Model 2	-5.705 (-6.028 to -5.381)	-6.008 (-6.601 to -5.415)	-5.974 (-6.569 to -5.378)	0.35	0.55
**Total brain volume, %**					
Model 1	-3.355 (-3.489 to -3.221)	-3.689 (-3.934 to -3.444)	-3.414 (-3.659 to -3.169)	0.31	0.49
Model 2	-3.363 (-3.496 to -3.229)	-3.661 (-3.906 to -3.416)	-3.415 (-3.661 to -3.169)	0.37	0.51

Abbreviations: CIs, confidence intervals; VAS, visual analogue scale; FDR, false discovery rate.
Values where significant differences were observed are shown in bold (p<0.05).
Values were calculated as follows: ([regional brain volume in 2017 - regional brain volume in 2012] / regional brain volume in 2012) × 100 (%). Regional brain volumes were calculated as the sum of the left and right sides.
Model 1: Adjusted for age and sex.
Model 2: Adjusted for age, sex, education attainment, marital status, hypertension, diabetes, serum total cholesterol level, body mass index, current smoking, current drinking, regular exercise, cerebrovascular lesions on magnetic resonance imaging, activities of daily living disability, and depressive symptoms.
An FDR Q-value <0.10 was defined as sufficient to verify the multiple comparisons.

Supplementary table 4. Adjusted standardized partial regression coefficients of volume changes in each brain region at 5 years after baseline according to the presence of chronic pain (*n* = 766), 2012-2017

Brain region	Adjusted standardized partial regression coefficients (95% CIs) of volume changes		*P* value	*q* value of FDR correction
	No chronic pain (*n* = 479)	Chronic pain (*n* = 287)		
**Ventrolateral prefrontal cortex, %**				
Model 1	(reference)	-0.1050 (-0.2516 to 0.0416)	0.16	0.22
Model 2	(reference)	-0.0924 (-0.2409 to 0.0561)	0.22	0.27
**Dorsolateral prefrontal cortex, %**				
Model 1	(reference)	-0.0968 (-0.2437 to 0.0501)	0.20	0.22
Model 2	(reference)	-0.0866 (-0.2355 to 0.0622)	0.25	0.28
**Orbitofrontal cortex, %**				
Model 1	(reference)	-0.1011 (-0.2477 to 0.0456)	0.18	0.22
Model 2	(reference)	-0.0939 (-0.2429 to 0.0551)	0.22	0.30
**Postcentral gyrus, %**				
Model 1	(reference)	**-0.2014 (-0.3478 to -0.0550)**	**0.007**	**0.03**
Model 2	(reference)	**-0.1909 (-0.3391 to -0.0428)**	**0.01**	**0.04**
**Insular cortex, %**				
Model 1	(reference)	-0.1328 (-0.2778 to 0.0121)	0.07	0.16
Model 2	(reference)	-0.1168 (-0.2632 to 0.0297)	0.12	0.22
**Thalamus, %**				
Model 1	(reference)	**-0.2220 (-0.3683 to -0.0756)**	**0.003**	**0.02**
Model 2	(reference)	**-0.2076 (-0.3562 to -0.0591)**	**0.006**	**0.03**
**Anterior cingulate cortex, %**				
Model 1	(reference)	**-0.2223 (-0.3674 to -0.0772)**	**0.003**	**0.03**
Model 2	(reference)	**-0.2159 (-0.3633 to -0.0684)**	**0.004**	**0.046**
**Posterior cingulate cortex, %**				
Model 1	(reference)	-0.1309 (-0.2775 to 0.0157)	0.08	0.13
Model 2	(reference)	-0.1158 (-0.2640 to 0.0325)	0.13	0.20
**Amygdala, %**				
Model 1	(reference)	**-0.1518 (-0.2955 to -0.0081)**	**0.04**	0.11
Model 2	(reference)	**-0.1582 (-0.3034 to -0.0130)**	**0.03**	**0.09**
**Hippocampus, %**				
Model 1	(reference)	-0.0831 (-0.2239 to 0.0576)	0.25	0.25
Model 2	(reference)	-0.0780 (-0.2201 to 0.0640)	0.28	0.28
**Total brain volume, %**				
Model 1	(reference)	-0.1323 (-0.2778 to 0.0132)	0.07	0.14
Model 2	(reference)	-0.1179 (-0.2639 to 0.0280)	0.11	0.25

Abbreviations: CIs, confidence intervals; FDR, false discovery rate.
Values where significant differences were observed are shown in bold (p<0.05).
Volume changes were calculated as follows: ([regional brain volume in 2017 - regional brain volume in 2012] / regional brain volume in 2012) × 100 (%). Regional brain volumes were calculated as the sum of the left and right sides.
Model 1: Adjusted for age and sex.
Model 2: Adjusted for age, sex, education attainment, marital status, hypertension, diabetes, serum total cholesterol level, body mass index, current smoking, current drinking, regular exercise, cerebrovascular lesions on magnetic resonance imaging, activities of daily living disability, and depressive symptoms.
An FDR Q-value <0.10 was defined as sufficient to verify the multiple comparisons.

Supplementary table 5. Multivariable-adjusted mean values of volume changes in each brain region at 5 years after baseline according to the pain intensity of chronic pain (*n* = 765), 2012-2017

Brain region	Multivariable-adjusted standardized partial regression coefficients (95% CIs) of volume changes, %			*P* for trend	*q* value of FDR correction
	No chronic pain (*n* = 479)	Chronic pain, VAS ≤39.4 mm (*n* = 143)	Chronic pain, VAS >39.4mm (*n* = 143)		
**Ventrolateral prefrontal cortex, %**	(reference)	-0.1525 (-0.3413 to 0.0363)	-0.0341 (-0.2239 to 0.1557)	0.45	0.55
**Dorsolateral prefrontal cortex, %**	(reference)	-0.1498 (-0.3390 to 0.0394)	-0.0252 (-0.2155 to 0.1650)	0.51	0.56
**Orbitofrontal cortex, %**	(reference)	-0.1686 (-0.3580 to 0.0208)	-0.0182 (-0.2086 to 0.1722)	0.52	0.52
**Postcentral gyrus, %**	(reference)	**-0.2520 (-0.4403 to -0.0637)**	-0.1305 (-0.3198 to 0.0587)	0.05	0.15
**Insular cortex, %**	(reference)	-0.1506 (-0.3368 to 0.0355)	-0.0867 (-0.2739 to 0.1005)	0.21	0.46
**Thalamus, %**	(reference)	**-0.2123 (-0.4012 to -0.0234)**	**-0.1980 (-0.3879 to -0.0082)**	**0.01**	**0.08**
**Anterior cingulate cortex, %**	(reference)	**-0.1941 (-0.3816 to -0.0065)**	**-0.2380 (-0.4266 to -0.0495)**	**0.006**	**0.06**
**Posterior cingulate cortex, %**	(reference)	-0.1708 (-0.3593 to 0.0177)	-0.0601 (-0.2496 to 0.1295)	0.30	0.54
**Amygdala, %**	(reference)	-0.1451 (-0.3298 to 0.0396)	-0.1697 (-0.3554 to 0.0160)	**0.04**	0.15
**Hippocampus, %**	(reference)	-0.0808 (-0.2614 to 0.0999)	-0.0717 (-0.2533 to 0.1099)	0.35	0.55
**Total brain volume, %**	(reference)	**-0.1976 (-0.3830 to -0.0122)**	-0.0346 (-0.2210 to 0.1518)	0.37	0.51

Abbreviations: CIs, confidence intervals; VAS, visual analogue scale; FDR, false discovery rate.
Values where significant differences were observed are shown in bold (p<0.05).
Volume changes were calculated as follows: ([regional brain volume in 2017 - regional brain volume in 2012] / regional brain volume in 2012) × 100 (%). Regional brain volumes were calculated as the sum of the left and right sides.
Values were adjusted for age, sex, education attainment, marital status, hypertension, diabetes, serum total cholesterol level, body mass index, current smoking, current drinking, regular exercise, cerebrovascular lesions on magnetic resonance imaging, activities of daily living disability, and depressive symptoms.
An FDR Q-value <0.10 was defined as sufficient to verify the multiple comparisons.

Supplementary table 6. Multivariable-adjusted mean values of volume changes in each left and right brain region at 5 years after baseline according to the presence of chronic pain (*n* = 766), 2012-2017

Brain region	Multivariable-adjusted standardized partial regression coefficients (95% CIs) of volume changes, %		*P* value	*q* value of FDR correction	*P* for hetero.	*q* value of FDR correction
	No chronic pain (*n* = 479)	Chronic pain (*n* = 287)				
**Ventrolateral prefrontal cortex, %**						
Left	(reference)	-0.0522 (-0.2007 to 0.0963)	0.49	0.58	0.44	0.73
Right	(reference)	-0.1138 (-0.2627 to 0.0352)	0.13	0.24		
**Dorsolateral prefrontal cortex, %**						
Left	(reference)	-0.0574 (-0.2063 to 0.0914)	0.45	0.56	0.14	0.35
Right	(reference)	-0.1115 (-0.2604 to 0.0373)	0.14	0.24		
**Orbitofrontal cortex, %**						
Left	(reference)	-0.0759 (-0.2252 to 0.0733)	0.32	0.42	0.67	0.83
Right	(reference)	-0.0961 (-0.2451 to 0.0528)	0.21	0.29		
**Postcentral gyrus, %**						
Left	(reference)	-0.1032 (-0.2522 to 0.0458)	0.17	0.27	0.07	0.22
Right	(reference)	**-0.2368 (-0.3844 to -0.0891)**	**0.002**	**0.03**		
**Insular cortex, %**						
Left	(reference)	0.0158 (-0.1320 to 0.1635)	0.83	**0.88**	**0.004**	**0.04**
Right	(reference)	**-0.2200 (-0.3664 to -0.0736)**	**0.003**	**0.03**		
**Thalamus, %**						
Left	(reference)	-0.1469 (-0.2956 to 0.0018)	0.05	0.13	0.69	0.76
Right	(reference)	**-0.1998 (-0.3483 to -0.0513)**	**0.008**	**0.06**		
**Anterior cingulate cortex, %**						
Left	(reference)	**-0.1711 (-0.3186 to -0.0236)**	**0.02**	**0.09**	0.72	0.72
Right	(reference)	**-0.1878 (-0.3362 to -0.0394)**	**0.01**	**0.07**		
**Posterior cingulate cortex, %**						
Left	(reference)	-0.0504 (-0.1990 to 0.0982)	0.51	0.56	0.16	0.32
Right	(reference)	-0.1480 (-0.2964 to 0.0004)	0.05	0.14		
**Amygdala, %**						
Left	(reference)	-0.1432 (-0.2896 to 0.0033)	0.06	0.12	0.59	0.84
Right	(reference)	-0.1279 (-0.2737 to 0.0180)	0.09	0.17		
**Hippocampus, %**						
Left	(reference)	0.0086 (-0.1336 to 0.1507)	0.91	0.91	**0.03**	0.14
Right	(reference)	**-0.1548 (-0.2985 to -0.0112)**	**0.03**	0.12		

Abbreviations: CIs, confidence intervals; FDR, false discovery rate; hetero., heterogeneity.
Values where significant differences were observed are shown in bold (p<0.05).
Volume changes were calculated as follows: ([regional brain volume in 2017 - regional brain volume in 2012] / regional brain volume in 2012) × 100 (%).
Values were adjusted for age, sex, education attainment, marital status, hypertension, diabetes, serum total cholesterol level, body mass index, current smoking, current drinking, regular exercise, cerebrovascular lesions on magnetic resonance imaging, activities of daily living disability, and depressive symptoms.
An FDR Q-value <0.10 was defined as sufficient to verify the multiple comparisons.
